# Sustained predation pressure may prevent the loss of anti‐predator traits from havened populations

**DOI:** 10.1002/ece3.11668

**Published:** 2024-07-10

**Authors:** Natasha D. Harrison, Ben L. Phillips, Adrian F. Wayne, Nicola J. Mitchell

**Affiliations:** ^1^ School of Biological Sciences University of Western Australia Crawley Western Australia Australia; ^2^ School of Molecular and Life Sciences Curtin University Bentley Western Australia Australia; ^3^ Biodiversity and Conservation Science, Department of Biodiversity Conservation and Attractions Manjimup Western Australia Australia

**Keywords:** agitation, *Bettongia penicillata ogilbyi*, escape behaviour, foraging, giving‐up density, prey naivete, vigilance

## Abstract

Conservation havens free of invasive predators are increasingly relied upon for fauna conservation, although havened populations can lose anti‐predator traits, likely making them less suitable for life ‘beyond the fence’. Sustaining low levels of mammalian predator pressure inside havens may prevent the loss of anti‐predator traits from havened populations. We opportunistically compared behavioural and morphological anti‐predator traits between four woylie (*Bettongia penicillata ogilbyi*) populations– one haven isolated from all mammalian predators, one haven containing a native mammalian predator (chuditch; *Dasyurus geoffroii*), and their respective non‐havened counterparts (each containing both chuditch and invasive predators). Havened woylies existing without mammalian predators were smaller (shorter hindfeet, smaller body weight) and less reactive (consumed more food from fox‐treated and control feeding stations, less agitated during human handling) than a non‐havened reference population. However, in the haven containing chuditch, we found no difference in behaviour or morphology compared to the adjacent non‐havened population. Across populations, anti‐predator responses tended to appear stronger at sites with higher predator activity, suggestive of an adaptive response across a gradient of predation pressure. Our findings suggest that maintaining mammalian predation pressure in conservation havens could be effective for preventing or slowing the loss of anti‐predator traits from these populations.

## Graphical abstract

Mammal populations in predator‐free havens can rapidly lose their anti‐predator traits. We investigate the role that predation from native predators inside havens may play in preventing the loss of these critical traits. We show that the presence of chuditch appears to have prevented the loss of behavioural and morphological anti‐predator traits from a havened population of woylies.

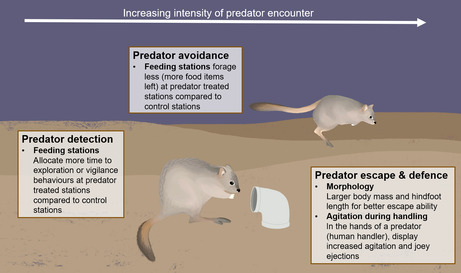

## 1 INTRODUCTION

Invasive predators are a leading cause of global biodiversity loss, implicated in the extinction of close to 150 bird, reptile and mammal species (Doherty et al., 2016). Invasive predators are also near‐impossible to eradicate from a landscape (Evans et al., 2022), so conservation managers are increasingly turning to predator‐free havens (e.g. fenced reserves or offshore islands) to protect and recover imperilled populations (Keitt et al., 2011; Legge et al., 2023; Russell et al., 2015). Havens are very effective – without large mammalian predators, many populations grow rapidly to reach high densities and havens have prevented the extinction of multiple mammal species (Legge et al., 2018). But havens are not without drawbacks (Read et al., 2023). In the absence of predators, havened populations can lose their anti‐predator responses (Harrison, Phillips, Mitchell, et al., 2023; Jolly et al., 2018; Messler et al., 2007; Muralidhar et al., 2019) either through rapid evolution, by plastic trait shifts, or a combination of the both mechanisms (Harrison, 2024). This can often lead to lower survival when translocated to areas where predators persist (Harrison et al., in review‐a; Ross et al., 2019). This has important implications for conservation as translocations are being increasingly relied upon to recover and protect imperilled populations. For havens to act as effective sources of animals to establish new and supplement existing populations, we need to halt or reduce the rate at which anti‐predator traits are lost from havened populations.

Inside havens, in the absence of predation pressure, predation‐driven selection is relaxed and anti‐predator traits that are no longer functionally beneficial will gradually erode from the population (Lahti et al., 2009). Further still, in havened populations experiencing limited resources, anti‐predator traits may incur a cost and be actively selected against. Without predation, resource competition can become the dominant selection pressure and traits that once served to evade predators, like vigilance behaviour or large body size, will disadvantage individuals during competition for resources (Jolly & Phillips, 2020). Under these conditions, we can expect anti‐predator traits to be rapidly winnowed from the population by natural selection. Traits acquired through experiential learning may also be lost without predator encounters from which to learn (Blumstein, 2006). Despite the complexity of these mechanisms, conservation managers can manipulate conditions inside havens to favour beneficial traits. Consistent with these theories, we would expect sustained predation pressure to prevent the loss of anti‐predator traits from havened populations by providing the necessary predator encounters for learned behaviours, by keeping population density below carrying capacity and by applying selection pressures to favour suitable evolved traits (Harrison, 2024; Moseby et al., 2023). Here, we explore this idea using a threatened Australian marsupial, the woylie (brush‐tailed bettong *Bettongia penicillata ogilbyi*), as a study system.

More woylies have been translocated for conservation than any other native mammal in Australia, with over 45 reintroductions, approximately 70% of which have failed likely due to predation (Yeatman & Groom, 2012). Once found across most of southern and semi‐arid Australia, indigenous woylie populations are now restricted to two regions within their former range; the Upper Warren, and the Dryandra region in Western Australia. In both of these regions, insurance populations of woylies occur inside conservation havens, as do remnant indigenous populations that have been exposed to invasive predators, feral cats (*Felis catus*) and red foxes (*Vulpes vulpes*), for over 100 years (Fairfax, 2019; Koch et al., 2015). The Perup Sanctuary in the Upper Warren region contains a stable population of woylies that is isolated from all mammalian predators (Harrison et al., 2024) and has demonstrated a steady decline in behavioural and morphological anti‐predator traits since its establishment (Harrison, Phillips, Mitchell, et al., 2023). However, the sanctuary in the Dryandra region is effectively free of invasive predators, but contains a population of a native mesopredator, the chuditch (*Dasyurus geoffroii*). We investigate whether sustained predation pressure on woylies by chuditch inside the Dryandra sanctuary may have maintained anti‐predator traits in this havened population.

In this observational study, we compare a suite of behavioural and morphological anti‐predator traits between havened and non‐havened woylies in both the Upper Warren and Dryandra regions. We explore whether there is evidence consistent with a haven effect on anti‐predator responses, and whether there is any evidence to suggest that sustained predation pressure in the Dryandra sanctuary has slowed or prevented the loss of anti‐predator traits from this havened population. Specifically, we compare (1) body size (body weight and relative hindfoot length), (2) agitation during human handling, (3) joey ejection rates and (4) behaviour at feeding stations treated with predator‐cues (giving‐up densities, proportion of time allocated to foraging and vigilance) across each of the four populations. These metrics represent aspects of predator escape, defence and detection/avoidance respectively. If there is a loss of anti‐predator traits from havened populations depending on their level of exposure to mammalian predators, then we hypothesise that predator‐free havened woylies will be smaller than non‐havened woylies in the Upper Warren (as larger size facilitates faster escape; Tay et al., 2021), but that there will be less or no difference in size between havened and non‐havened woylies in Dryandra (where the haven contains chuditch). Similarly, that havened woylies in the Upper Warren will have reduced anti‐predator behaviour compared with non‐havened woylies (lower levels of agitation, less joey ejections, lower giving‐up‐densities, less time allocated to vigilance at predator‐treated feeding stations), but there will be little difference in the behaviour of havened and non‐havened woylies in Dryandra.

## 2 METHODS

### 2.1 Study species

Woylies (brush‐tailed bettong *Bettongia penicillata ogilbyi*), are a medium‐sized (males ~1300 g; females ~1100 g; Harrison et al., 2024) Australian marsupial listed as Endangered under the Environment Protection and Biodiversity Conservation (EPBC) Act 1999. As ecosystem engineers, they play a critical role in their environment as bioturbators and seed dispersers (Garkaklis et al., 2004; Murphy et al., 2005). Woylie joeys remain in their mother's pouch until independence at approximately 150 days post‐partum (Thompson et al., 2015). During this time, if woylie mothers perceive a threat, they are known to eject their joeys as a distraction technique to facilitate their own escape (Thompson et al., 2015).

### 2.2 Study populations

**FIGURE 1:**
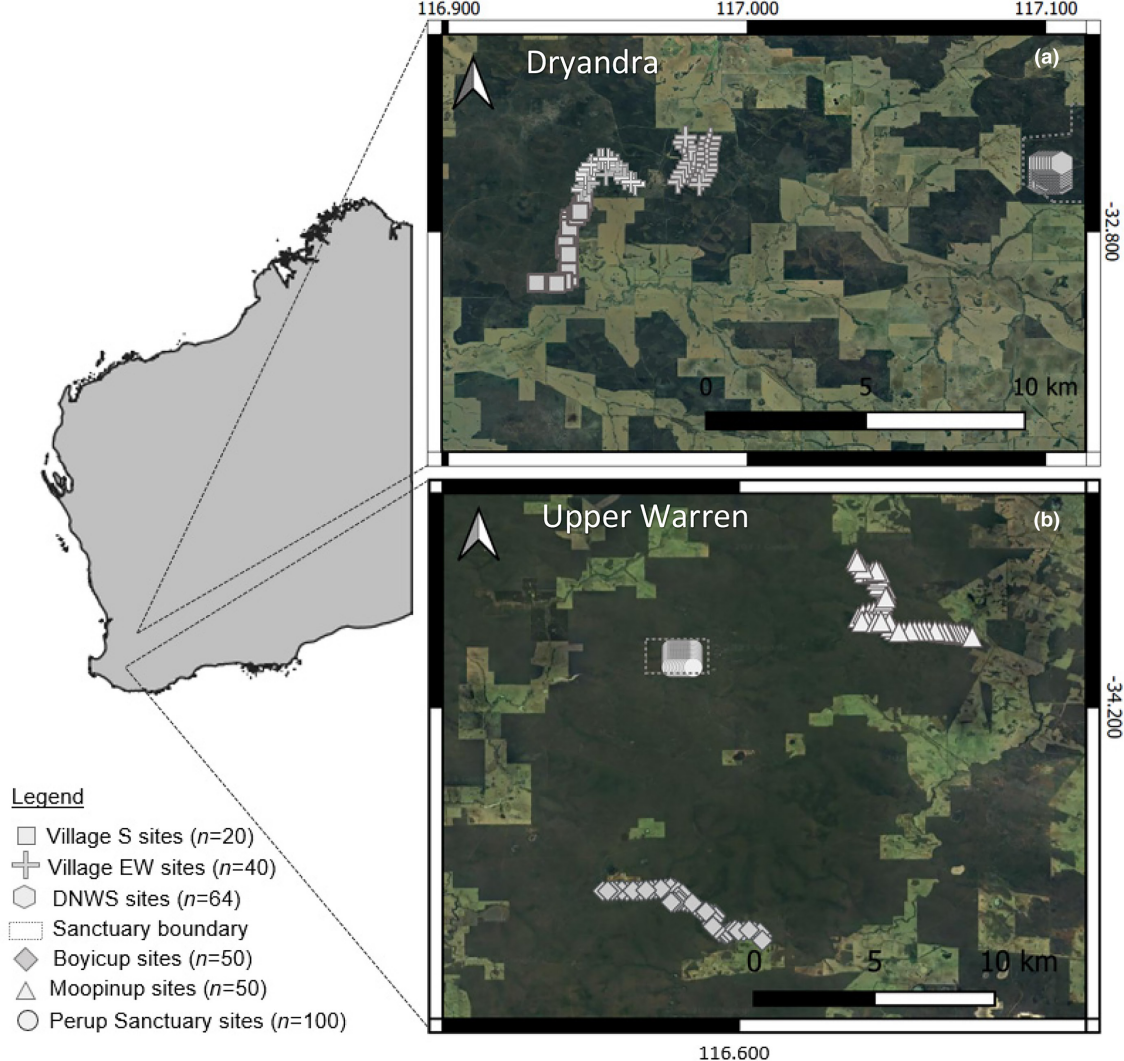
Map of havened and non‐havened sites within Dryandra National Park in the Dryandra Region (a; Village South, Village East and West in the main forest block, and Dryandra Numbat Woylie Sanctuary, DNWS) and Upper Warren Region (b; Boyicup, Moopinup and Perup Sanctuary), in Western Australia. Each point represents a cage trap location, and conservation haven boundaries are shown by grey dotted lines.

**FIGURE 2:**
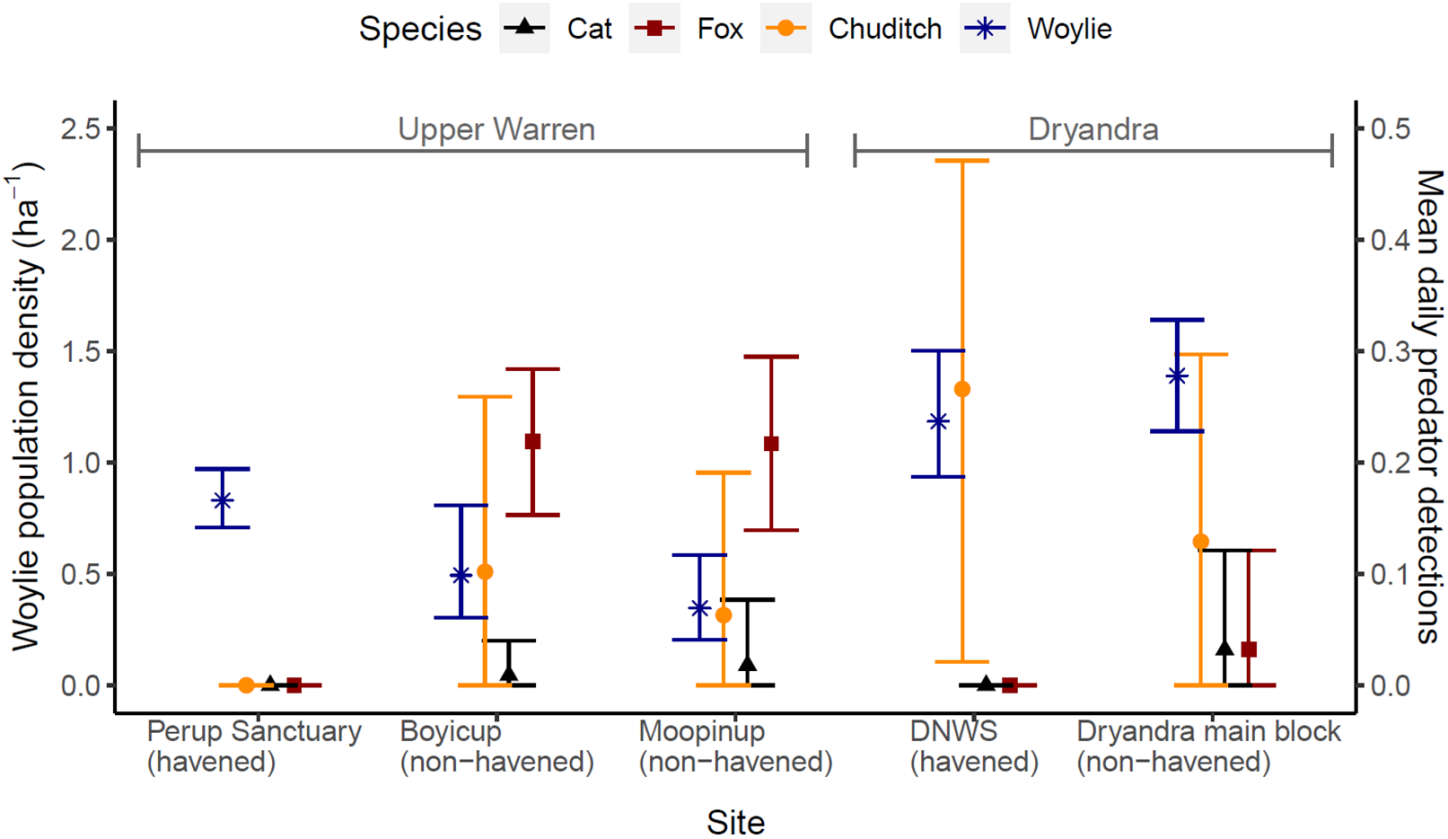
Woylie density (blue asterisk; left axis) and predator activity levels (right axis) among sites. Error bars represent 95% confidence intervals. Camera deployment to detect predators varied between regions (on roads in Upper Warren, off‐track in Dryandra) and so predator activity is not directly comparable between regions. Details of sampling design and data origin for each site can be found in Table S1.

**FIGURE 3:**
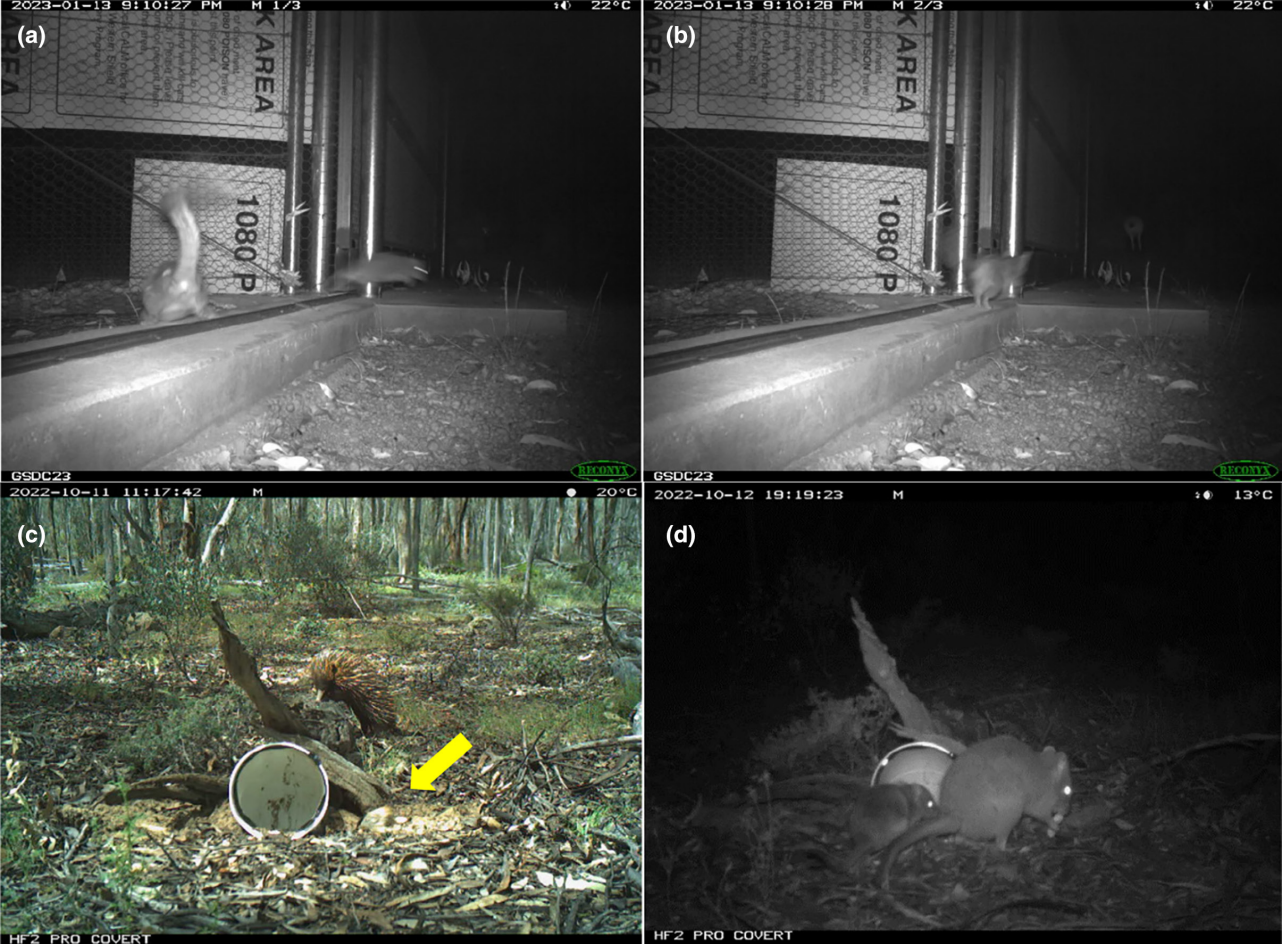
Motion‐sensor camera trap images of (a, b) a chuditch (*Dasyurus geoffroii*) hunting a woylie (*Bettongia penicillata ogilbyi*) inside the Dryandra Sanctuary (these images are one second apart and show the chuditch in rapid pursuit of a fleeing woylie; photo credit: DBCA Wheatbelt Region) and examples of PVC pipe feeding stations (with fox cue visible to the right of the entrance shown by yellow arrow) visited by (c) an echidna (*Tachyglossus aculeatus*) and (d) a woylie with her joey.

Here, we examined havened and non‐havened woylie populations in two regions: the Upper Warren and Dryandra (Figure 1) which allows for two replicates of a paired comparison to be made, where both havened populations generally share the same evolutionary history and environment as their non‐havened counterparts. In the Upper Warren, the 423 ha Perup Sanctuary (Figure 1b) was established in 2010 and since then, the resident woylies have been isolated from all native and invasive mammalian predators (Harrison et al., 2024). In Dryandra, the 1000 ha Dryandra Numbat Woylie Sanctuary (DNWS; Figure 1a) was established in 2016, and while it excludes large mammalian invasive predators, it contains a population of a Dasyurid predator, the chuditch (or western quoll, a marsupial carnivore), that coexists with the woylies inside the sanctuary (Figure 2). Despite their small size (males ~1300 g; females ~900 g; Serena & Soderquist, 1995), chuditch are considered potentially capable of predating adult woylies – chuditch have been observed hunting woylies (Figure 3), their DNA has been detected on woylie carcasses (Marlow et al., 2015; Wayne et al., 2011, 2013) and chuditch are thought to predate adults of the similar‐sized burrowing bettong (boodie, *Bettongia lesueur*; Stepkovitch et al., 2023). Both havened populations have remained exposed to predation by aerial and reptilian predators, though we assume that anti‐predator responses to these species differ from those needed to respond to mammalian predators (e.g. varanids are pursuit predators, whereas foxes and cats tend to be ambush hunters; Tay et al., 2021), and that continuity of predation pressure by birds and reptiles would not be sufficient to influence the loss of mammalian‐specific anti‐predator traits. The non‐havened control populations we sampled within each region include Boyicup and Moopinup in the Upper Warren, and Village South and Village East/West within the Dryandra main forest block (Figure 1).

### 2.3 Field methods

**FIGURE 4:**
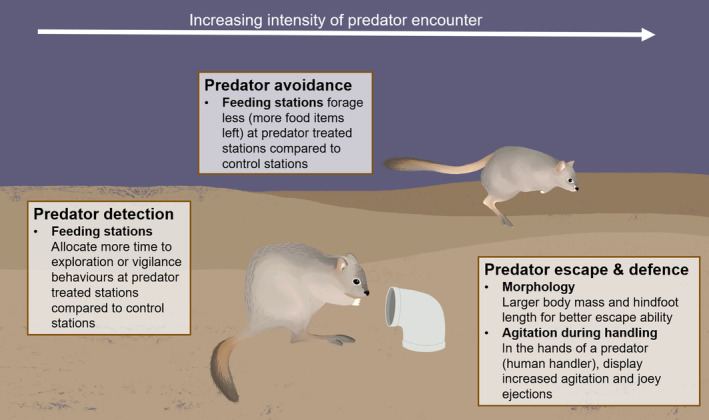
Schematic detailing our expectations of appropriate anti‐predator responses to evade mammalian predators from the metrics quantified in this study. Illustrations by Stephanie Mason.

We quantified a variety of behavioural and morphological anti‐predator traits related to predator detection, avoidance, escape and defence (Figure 4).

Trapping to monitor the size and health of all woylie populations was conducted using standard cage traps (details in Table S1). In brief, traps were opened for four nights and baited with a mix of rolled oats, peanut butter and sardines. When woylies were captured, they were given a microchip for individual identification (Microchips Australia), and we recorded their sex, weight, pes length, head length and reproductive status (including any ejections of pouch young by females). We also quantified their anti‐predator behaviour using an assay that measures agitation in the presence of a large unfamiliar ‘predator’, the human handler (described by Harrison et al., 2022). This assay quantifies agitation at five points: when the handler (1) approaches the trap, (2) places the capture bag over the trap, (3) opens the trap door, (4) has the animal in the capture bag before handling and (5) handles the animal for processing.

To observe how woylies forage in the presence of predator cues, we deployed feeding stations (90° 160 mm PVC storm pipe elbows as described in Harrison, 2024; Figure 3c,d) spaced 200 m apart, where each station was paired with a motion triggered camera trap (Reconyx Hyperfire 2, Swift Enduro; Outdoor Cameras, Australia) constantly recording and programmed to record 30 s videos when triggered. The aim of these stations was to measure giving‐up‐densities (the food density at which the animal chose to leave the foraging patch, whereby greater amounts of food left behind suggests more risk perceived; Brown et al., 1988; Harrison, Phillips, Hemmi, et al., 2023), and to observe how woylies behave in the presence of predator cues (including foraging, grooming, vigilance, see Table 1). Each station was treated with either a predator cue (a piece of fox tail plus an anal gland secretion to maximise pungency) or a control cue (cotton wool). In an effort to ensure that the fox tail predator cues smelled realistic, tails and respective anal glands were removed from fox carcasses within 3 h of death, and immediately frozen at −18°C until deployment. Stations were deployed for 3 days at a time (to ensure predator cues remained pungent, and to avoid habituation to the stations and cues) and rebaited each morning to provide a total of seven shelled peanuts mixed into a soil matrix in the station each day. Treatment type and camera model were randomly distributed across the landscape at each site. We deployed 180 station nights in Dryandra (havened *n* = 90 and non‐havened *n* = 90) and 408 station nights in the Upper Warren (havened *n* = 120 and non‐havened *n* = 288).

**TABLE 1:** Ethogram of woylie (*Bettongia penicillata ogilbyi*) behaviours scored from camera trap video footage of individuals visiting predator‐treated feeding stations in the Upper Warren and Dryandra regions, Western Australia.

Category	Behaviour	Type	Description
Vigilance	Quadrupedal	State	On all four legs, looking around or sniffing the air
	Bipedal	State	Any time the animal is elevated on their two hindlegs
Foraging	Forage	State	Nose to ground, sniffing/scrabbling/eating/digging
	Eat (dine in)	Count	Eat peanut in field of view
	Eat (take away)	Count	Carry peanut out of field of view
Exploration	Sniff station/cue	State	Sniffing around the predator cue or PVC pipe (i.e., in front of station or facing station within ~20 cm)
	Entry to station	State	Front legs and head inside the PVC pipe
Movement	Locomotion	State	Walking or running
Other	Grooming	State	Grooming or scratching
	Competition	State	At least one additional animal (of any species) present in the field of view
	Aggression	State	Any negative interaction with another animal
	Tail swish	Count	Waving tail in a swiping motion
	Roll over	State	Rolling onto back or side; a submissive behaviour
	Other	State	Any behaviours not mentioned above

To obtain behavioural metrics from the camera trap data, video footage was scored by three observers using the open‐source BORIS software (Behavioural Observation Research Interactive Software; Friard & Gamba, 2016). To ensure accuracy, each observer initially scored an overlap of footage, and an inter‐rater reliability test was performed through the BORIS software at 300 time points to check for agreeance in scoring, which was >0.8 among all three observers. Videos were randomly distributed among observers who remained blind to treatment and sites. We scored all behaviours from footage within an hour of the first woylie visit per station per night (Table 1).

### 2.4 Statistical analysis

All statistical analyses were conducted in the R environment, version 4.0.3 (R Core Team, 2020). We used generalised and linear models (with random effects where necessary; mixed effects models) from the packages ‘lme4’ (Bates et al., 2015) and ‘glmmTMB’ (Brooks et al., 2017) detailed below. Across all model types, we used likelihood ratio tests to evaluate the significance of each parameter (*α* = 0.05), and tested model fit using the ‘DHARMa’ package (Hartig, 2022). Interaction effects were explored by conducting Tukey post‐hoc pairwise comparisons using the package ‘emmeans’ (Lenth, 2024).

#### 2.4.1 Morphology

To test for differences in morphology between havened and non‐havened populations among regions, we built two Gaussian linear models investigating body weight and relative pes length (pes length relative to the size of the animal by accounting for head length). In each model, we tested for the effects of sex (male or female), and the interaction between population type (havened or non‐havened) and region (Dryandra or Upper Warren). Both response variables were log transformed to ensure linearity, and in the pes model, we included log(head length) as a predictor variable to control for changes in pes length as a result of body size.

#### 2.4.2 Behaviour

We built five binomial generalised linear mixed effects models to explore differences in giving‐up‐densities (binary: 1 if all peanuts consumed), mode of consumption (proportion of peanuts consumed dine‐in as opposed to take‐away), and proportion of time allocated to foraging, vigilance and exploration behaviours between havened and non‐havened populations among regions. In each model, we tested for the effects of deployment night (1, 2 or 3), treatment (fox or control), woylie population density, and the interaction between region (Dryandra or Upper Warren) and population type (havened or non‐havened), with station number as a random effect. In the models exploring foraging, vigilance and exploration behaviour, we also included observer as a random effect.

To compare agitation scores among populations, we built a Gaussian linear mixed effects model testing for the effects of sex, number of previous captures, the interaction effect between region and haven, including handler and individual identity as random effects. As agitation scores are not obviously normally distributed, we carefully examined model residuals; the residuals were symmetrical, homoscedastic and approximately normally distributed. To compare joey ejections between populations, we limited the data to females with pouch young only. While we had aimed to explore the effect of the interaction between haven and region on joey ejection probability (0 or 1), the data were too zero inflated to build a robust model, so instead we report this using a contingency table.

#### 2.4.3 Effect sizes

As there are likely to be confounding factors and different selective pressures acting within each region, we calculated an effect size, Cohen's d (Cohen, 1988), using the ‘effsize’ package (Torchiano, 2020) to compare differences in each behavioural and morphological metric between havened and non‐havened populations within each region.

## 3 RESULTS

### 3.1 Morphology

**FIGURE 5:**
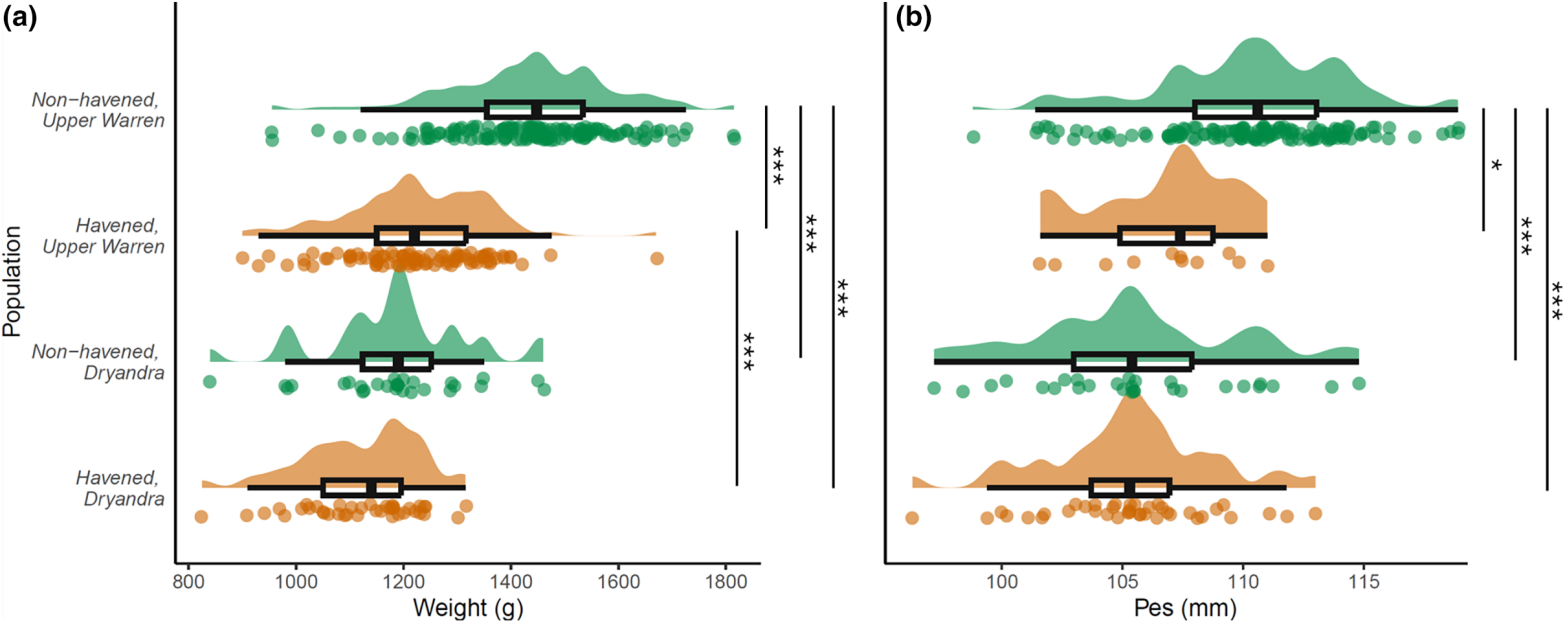
Rain plot showing weight (a) and pes length (b) of havened (orange) and non‐havened (green) woylie (*Bettongia penicillata ogilbyi*) populations from the Upper Warren and Dryandra regions in Western Australia. Asterisks represent the results of Tukey post‐hoc pairwise comparisons between groups.

We compared morphometrics from 75 Dryandra woylies (havened *n* = 38, non‐havened *n* = 37) and 232 Perup woylies (havened *n* = 87, non‐havened *n* = 145). There was a significant interaction between havening and region. Non‐havened woylies from the Upper Warren were heavier than havened (*t*(296) = −12.81, *p* < .001) and non‐havened woylies (*t*(296) = −8.80, *p* < .001) from Dryandra (Figure 5; Table 2). Within the Upper Warren Region, non‐havened woylies were heavier (mean = 1440 g) than havened woylies (mean = 1225 g) (*t*(296) = −10.80, *p* < .001). Similarly, non‐havened Dryandra woylies were slightly heavier (mean = 1185 g) than the havened Dryandra woylies (mean = 1120 g; Figure 5; Table 2).

**TABLE 2:** Effect of population and region on weight, relative pes length and agitation behaviour in woylies (*Bettongia penicillata ogilbyi*) from the Upper Warren and Dryandra regions in Western Australia.

Variable	Weight (g)				Relative pes (mm)				Agitation score			
	Effect	SE	Df	*p*‐value	Effect	SE	Df	*p*‐value	Effect	SE	Df	*p*‐value
Intercept	7.007	0.020	–	–	3.733	0.225	–	–	4.672	0.424	–	–
Sex_Male	0.013	0.013	1	.323	0.001	0.005	1	.886	0.422	0.224	1	.058
Haven_NonHavened	0.053	0.027	1	**<.001**	−0.002	0.008	1	.051	0.345	0.501	1	**<.001**
Region_UpperWarren	0.092	0.021	1	**<.001**	0.013	0.012	1	**<.001**	0.374	0.519	1	**<.001**
Log(Head)	–	–	–	–	0.209	0.051	1	**<.001**	–	–	–	–
PreviousCaptures	–	–	–	–	–	–	–	–	0.001	0.004	1	.684
Haven_NonHavened: Region_UpperWarren	0.105	0.031	1	**<.001**	0.028	0.013	1	**.039**	2.367	0.619	1	**<.001**

*Note*: The slope, standard error (SE) and degrees of freedom (df) for each variable in the final model is reported (with levels of a particular variable indicated after the underscore), as is the *p*‐value resulting from likelihood ratio tests of the final model with and without each respective variable (*p* < .05 are shown in bold).

Relative pes lengths showed a similar pattern with a significant interaction between havening and region. Non‐havened woylies in the Upper Warren had longer pes compared to havened (*t*(296) = −12.81, *p* < .001) and non‐havened woylies in Dryandra (Figure 5; Table 2). Non‐havened woylies had longer pes relative to havened woylies in the Upper Warren (*t*(194) = −2.45, *p* = .07; mean = 110 mm and 107 mm, respectively), but in Dryandra there was no difference in pes length between havened and non‐havened woylies (mean = 105 mm; Figure 5; Table 2). There was no effect of sex on weight or pes length (Table 2).

### 3.2 Behaviour

**FIGURE 6:**
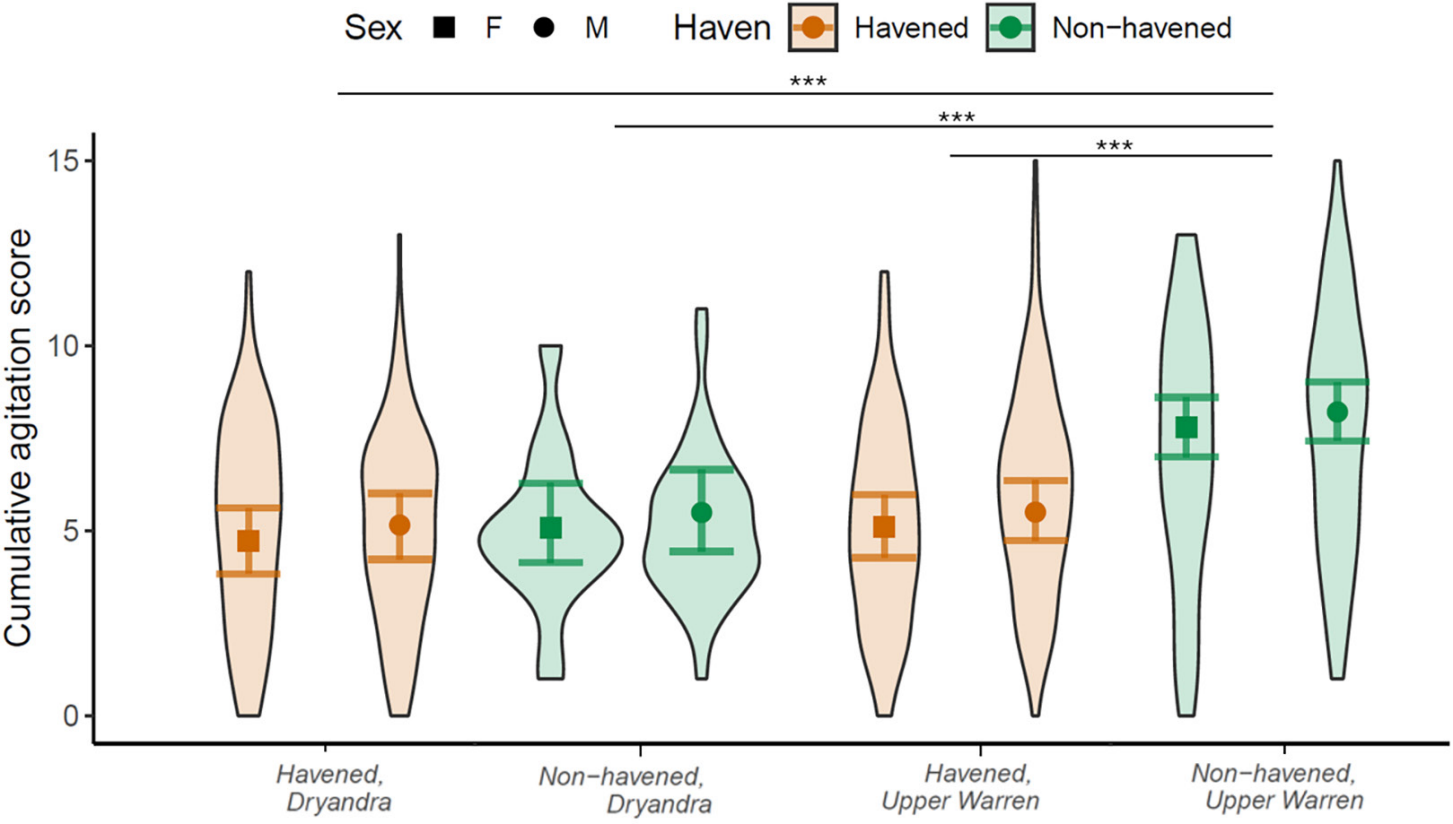
Agitation scores of male (circle) and female (square) havened (orange, left) and non‐havened (green, right) woylies (*Bettongia penicillata ogilbyi*) from the Upper Warren and Dryandra regions in Western Australia. Raw data is illustrated by violin plots and model predictions are shown by points with error bars indicating standard error. Asterisks represent the results of Tukey post‐hoc pairwise comparisons between groups.

Agitation scores were higher in non‐havened woylies (mean = 7.45) compared to havened woylies (mean = 5.57) in the Upper Warren (*t*(468.6) = −7.44, *p* < .001), yet agitation scores were comparable between havened (mean = 5.05) and non‐havened woylies (mean = 5.10) in Dryandra (Figure 6). Non‐havened woylies in the Upper Warren had higher agitation scores than havened (*t*(113.5) = −6.41, *p* < .001) and non‐havened (*t*(192.1) = −4.25, *p* < .001) woylies in Dryandra. Agitation scores were slightly higher in males compared to females but were not affected by the number of times an individual had been captured previously (Table 2).

There were more ejections from non‐havened woylies (17%) compared to havened woylies (0%) in the Upper Warren, but the proportion of females that ejected was comparable between havened (9%) and non‐havened (8%) woylies in Dryandra (Table 3). It is important to note, however, that this does not account for potential confounding factors, such as the size of the joey or the capture history of the mother (known to impact ejection probabilities; Harrison, Phillips, Mitchell, et al., 2023).

**TABLE 3:** Number of joey ejections by female woylies (*Bettongia penicillata ogilbyi*) carrying pouch young from the Upper Warren and Dryandra regions in Western Australia.

Region	Population type	Ejected	Not ejected
Dryandra	Havened	2	21
	Non‐havened	6	72
Upper Warren	Havened	0	52
	Non‐havened	9	44

We scored more than 700 h of footage of woylies at feeding stations, resulting in 7368 individual behaviours. There was no effect of treatment, havening, region or population density on the proportion of time allocated to exploration, foraging and vigilance by woylie populations (Table 4).

**TABLE 4:** Effect of population and region on the probability of all peanuts being eaten, the proportion of peanuts taken away and the proportion of time allocated to foraging, vigilance and exploration by woylies (*Bettongia penicillata ogilbyi*) at predator‐treated feeding stations in the Upper Warren and Dryandra regions in Western Australia.

Variable	GUD				Proportion of peanuts take‐away				Foraging				Vigilance				Exploration			
	Effect	SE	Df	*p*‐value	Slope	SE	Df	*p*‐value	Slope	SE	Df	*p*‐value	Slope	SE	Df	*p*‐value	Slope	SE	Df	*p*‐value
Intercept	−4.737	7.060	–	–	−3.713	5.930	–	–	−0.266	0.843	–	–	−0.202	4.696	–	–	−2.097	2.940	–	–
Region_UpperWarren	7.428	2.735	1	**.001**	1.049	1.847	1	.482	−0.783	1.001	1	.921	−0.115	1.534	1	.691	0.876	0.905	1	.611
Haven_NonHavened	−4.645	1.780	1	**<.001**	−0.052	1.186	1	.612	0.544	0.650	1	.852	0.096	1.062	1	.528	−0.572	0.595	1	.600
Night	1.847	0.307	1	**<.001**	0.087	0.248	1	.726	0.137	0.101	1	.172	0.001	0.207	1	.997	−0.191	0.115	1	.097
WoylieDensity	3.529	5.910	1	.547	2.659	4.963	1	.588	−0.589	0.936	1	.532	−0.709	3.885	1	.856	2.249	2.466	1	.360
Treatment_Fox	−1.625	0.729	1	**.014**	−0.147	0.396	1	.711	0.160	0.205	1	.576	0.415	0.338	1	.436	−0.174	0.188	1	.355
Haven_NonHavened: Region_UpperWarren	−2.067	1.859	1	.979	0.730	2.300	1	.807	−0.365	1.662	1	.413	−0.173	3.235	1	.836	1.236	1.493	1	.409

*Note*: The slope, standard error (SE) and degrees of freedom (df) for each variable in the final model is reported (with levels of a particular variable indicated after the underscore), as is the *p*‐value resulting from likelihood ratio tests of the final model with and without each respective variable (*p* < .05 are shown in bold).

**FIGURE 7:**
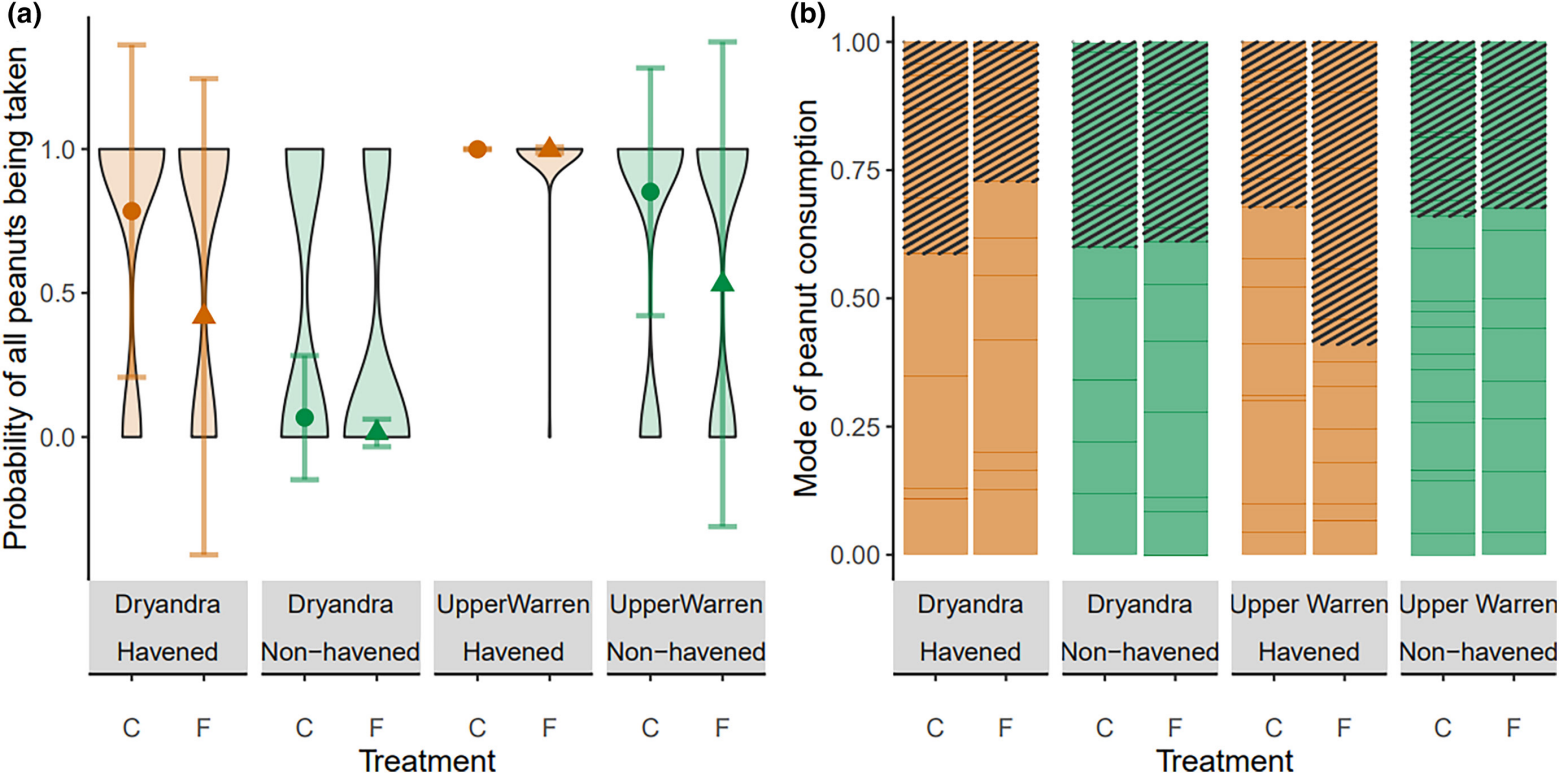
Probability of all seven peanuts being eaten ± SE (a; violin plots show raw data) and proportion of peanuts eaten dine‐in (striped) or take‐away (no pattern) (b) by havened (orange) and non‐havened (green) woylie (*Bettongia penicillata ogilbyi*) populations from two Western Australian regions at feeding stations treated with fox cues (triangles; F) or control stations (circles; C).

In contrast, giving‐up densities varied between treatments and among populations. Woylies from the Perup Sanctuary population ate 100% of peanuts from all stations (Figure 7a). In the other three cohorts, the probability of all peanuts being eaten was higher at control stations compared to fox‐treated stations (Figure 7a). The probability of all peanuts being eaten was higher in the Upper Warren (mean = 0.92) compared to Dryandra (mean = 0.15), and higher in havened populations (mean = 0.65) compared to non‐havened populations (mean = 0.42; Table 4). Across all populations, probability of all peanuts being eaten increased with increasing deployment nights, and woylie population density did not affect the probability of consumption (Table 4). There was no effect of region, deployment night, havening, treatment, or woylie density on the mode of peanut consumption (Table 4; Figure 7b). There was a weak (*p* = .09) negative effect of deployment night on proportion of time allocated to exploration behaviour, but deployment night did not affect foraging or vigilance (Table 4).

### 3.3 Effect sizes

Effect sizes revealed that differences in weight, pes length, agitation behaviour and joey ejections between woylies from havened and non‐havened populations were greater in the Upper Warren (mean = 0.92) compared to Dryandra (mean = 0.15; Table 5). Differences in giving‐up densities, foraging, vigilance and exploration at fox and control stations were mostly negligible and comparable across havened and non‐havened populations across regions (Table 5).

**TABLE 5:** Effect sizes of behavioural and morphological differences between havened and non‐havened woylie (*Bettongia penicillata ogilbyi*) populations in the Upper Warren and Dryandra regions of Western Australia. Small effects are coloured in purple (<0.5), moderate effects in yellow (<0.9) and large effects in green. Effects where the 95% confidence interval does not cross zero are shown in bold.

Trait	Level	Region	Effect size (95% CI)
Weight	Individual	Dryandra (Havened/Non‐havened)	**0.528 (0.025–1.031)**
		Upper Warren (Havened/Non‐havened)	**1.495 (1.196–1.793)**
Pes	Individual	Dryandra (Havened/Non‐havened)	0.067 (−0.427–0.562)
		Upper Warren (Havened/Non‐havened)	**0.940 (0.312–1.569)**
Agitation	Individual	Dryandra (Havened/Non‐havened)	0.017 (−0.247–0.282)
		Upper Warren (Havened/Non‐havened)	**0.629 (0.424–0.835)**
Joey ejections	Individual	Dryandra (Havened/Non‐havened)	0.003 (−0.434–0.508)
		Upper Warren (Havened/Non‐havened)	**0.630 (0.234–1.027)**
GUD	Population	Dryandra Havened (Fox/Control)	0.217 (−0.204–0.637)
		Dryandra Non‐havened (Fox/Control)	0.199 (−0.221–0.619)
		Upper Warren Havened (Fox/Control)	0.183 (−0.180–0.545)
		Upper Warren Non‐havened (Fox/Control)	**0.234 (0.001–0.467)**
Foraging	Population	Dryandra Havened (Fox/Control)	−0.213 (−0.560–0.133)
		Dryandra Non‐havened (Fox/Control)	−0.413 (−0.937–0.108)
		Upper Warren Havened (Fox/Control)	−0.020 (−0.354–0.314)
		Upper Warren Non‐havened (Fox/Control)	0.090 (−0.247–0.427)
Vigilance	Population	Dryandra Havened (Fox/Control)	0.017 (−0.328–0.363)
		Dryandra Non‐havened (Fox/Control)	−0.006 (−0.524–0.511)
		Upper Warren Havened (Fox/Control)	**−0.814 (−1.161** to **−0.467)**
		Upper Warren Non‐havened (Fox/Control)	−0.167 (−0.504–0.170)
Exploration	Population	Dryandra Havened (Fox/Control)	0.137 (−0.209–0.483)
		Dryandra Non‐havened (Fox/Control)	0.271 (−0.249–0.791)
		Upper Warren Havened (Fox/Control)	0.262 (−0.072–0.598)
		Upper Warren Non‐havened (Fox/Control)	−0.071 (−0.407–0.266)

## 4 DISCUSSION

We opportunistically explored whether there was evidence that native mammalian predation pressure inside a haven free of invasive predators could prevent the loss of anti‐predator traits. We found that differences in behaviour and morphology between havened and non‐havened woylie populations in the Upper Warren, where the haven has been isolated from all mammalian predators for 11 years, were far greater than those in Dryandra, where the 7‐year‐old haven has maintained a population of a native predator. In most cases, there were no discernible differences in behaviour and morphology between havened and non‐havened populations in Dryandra. Our findings are therefore consistent with the idea that the presence and presumed predation pressure from chuditch inside the Dryandra haven may be preventing the loss of anti‐predator traits in the resident woylies.

Although we effectively only sampled two replicates, our findings align with those of multiple existing studies (Bannister et al., 2018; Jolly et al., 2018; Muralidhar et al., 2019). Specifically, the havened population void of any mammalian predation pressure in Perup Sanctuary has reduced anti‐predator abilities. This phenomenon has been documented globally, and across taxa, for example, in deer mice (*Peromyscus maniculatus*) in the United States of America (Orrock, 2010), tammar wallabies (*Macropus eugenii*; Blumstein et al., 2004) and South Island Robins (*Petroica australis australis*; Muralidhar et al., 2019) in New Zealand, and common brushtail possums (*Trichosurus vulpecula*; Bannister et al., 2018) and northern quolls (*Dasyurus hallucatus*; Jolly et al., 2018) in Australia. Although, there are limited studies exploring the survival consequences of these weakened traits (but see Bannister et al., 2018, 2021; Harrison et al., In review‐a; Ross et al., 2019), numerous studies that have imposed predation pressure inside havens have seen trait shifts in the direction predicted by theory (i.e., the reverse of what we found in Perup; increase in size and flightiness). For example, populations of predator naïve bilbies (*Macrotis lagotis*) and boodies (*Bettongia lesueur*) exposed to cats became larger and more reactive (Blumstein et al., 2019; Moseby et al., 2018), and survival following reintroduction to a site with foxes was positively correlated with body size in eastern bettongs (*Bettongia gaimardi;* Evans et al., 2021). Furthermore, Moseby et al. (2023) identified selection pressure operating on these morphological anti‐predator traits in boodies (selecting for larger individuals). This supports the theory that active selection from predation pressure inside the Dryandra sanctuary has maintained the traits that we found have weakened without it in Perup Sanctuary.

The presence of chuditch appears to be a potential mechanism to reduce the loss of anti‐predator traits from havened woylie populations, but we recognise that this is not a one‐size‐fits‐all solution. Although the Dryandra havened woylie population has been able to persist at high densities with some level of chuditch predation, this situation may not be suitable for all havened populations (Ross et al., 2019). For example, some havens may not be large enough to accommodate predators. A case in point is the Perup sanctuary, which at 423 ha is approximately one‐ third that of a male chuditch's home range (Serena & Soderquist, 1989). Moreover, not all havened populations may be capable of withstanding high levels of predation. When chuditch were reintroduced into Arid Recovery, a fenced haven in South Australia, larger species like boodies (burrowing bettongs; *Bettongia lesueur*) and bilbies (*Macrotis lagotis*) were able to persist, but in combination with other stressors, smaller species like the Shark Bay bandicoot (*Perameles bougainville*) and the greater stick‐nest rat (*Leporillus conditor*) experienced population declines (Stepkovitch et al., 2023). Hence, when considering imposing predation pressure inside havens, it is critical to carefully evaluate the level of predation that the population can sustain (Moseby et al., 2019; Stepkovitch et al., 2022). We suggest that future studies explore the thresholds of predation pressure at which havened populations may establish and persist, for example, using simulation studies to compare ‘harvest’ rates (e.g. via population viability analysis; Gibson Vega et al., 2023; Pacioni et al., 2019; Wilson et al., 2023).

Although, we find some evidence that predation pressure from chuditch prevented the loss of anti‐predator traits from the Dryandra havened woylie population, our results may be influenced by other factors. Potentially, the lack of decline in anti‐predator traits is a result of Dryandra experiencing less selection *against* these traits compared to Perup Sanctuary in the Upper Warren. Nonetheless, selection *for* anti‐predator traits imposed by chuditch has likely played an important role in maintaining these traits in the Dryandra havened population. In a related study, Harrison, Phillips, Mitchell, et al. (2023), showed that behavioural and morphological anti‐predator traits from the havened population in Perup Sanctuary declined in a predominantly linear fashion over 12 years. Although Dryandra had been isolated from invasive predators for 7 years, no erosion of anti‐predator traits was detectable across multiple metrics, despite the timeframe being sufficient for detecting declines. Both Dryandra populations and the Perup Sanctuary havened population exist at relatively high densities (Figure 2) and have slightly smaller body sizes compared to the non‐havened Perup population. Although we cannot solely attribute the lack of trait change to the presence of a native mammalian predator, the combination of potentially less resource competition (larger haven, isolated for less time) and ongoing predation pressure from chuditch seems be effectively maintaining anti‐predator traits in the Dryandra havened population.

Interestingly, we found considerable differences in behaviour and morphology between the two non‐havened populations. It is possible that anti‐predator responses may also be moderated in response to the level of predation pressure, and that we are observing the expression of anti‐predator traits along a gradient of predation pressure. Our on‐ground experience is that predator activity is higher in the Upper Warren compared to Dryandra, and though not directly comparable among sites (due to variation in camera deployment, see Table S1), the rates of predator activity presented here (Figure 2) are consistent with this. Our findings may indicate that populations of woylies display varying degrees of anti‐predator traits to commensurate variation in the risk of mammalian predation. This would be in line with theory suggesting that anti‐predator responses are a function of perceived predation risk (Lima & Bednekoff, 1999; Lima & Dill, 1990). Anti‐predator responses also appear to vary with increasing woylie population density. This mirrors the findings of a global meta‐analysis by Bolnick and Preisser (2005) which found that the strength of the negative effect of predation on prey varied with the level of competition. We suggest that woylie density may be partially driven by predation, and that the trade‐off between resource competition (indicated by high population density) and predator avoidance may be an important mediator of anti‐predator responses.

It is important to understand any potential survival consequences of havening to better inform species conservation at a regional or national scale (Harrison, Wayne, Mitchell, & Phillips, 2023). In the Australian context, there is increasing evidence demonstrating rapid shifts in anti‐predator responses when naïve populations of threatened mammals become exposed to invasive predators (Cunningham et al., 2019; Jolly et al., 2021; Moseby et al., 2023; Waaleboer et al., 2024). The apparent lack of trait loss from the Dryandra havened population, while promising, does not guarantee that individuals from this population will have comparable survival to their non‐havened counterparts when faced with feral cats and foxes. To this end, it would be valuable to evaluate the survival probabilities of both Dryandra populations in a common environment experiment, to determine whether the lack of difference in response translates to equal survival beyond the fence, when the havened individuals may face invasive predators they have never encountered. Indeed bilbies (*Macrotis* lagotis) exposed to chuditch appeared to be no better adapted to respond to feral cats than predator naïve bilbies (Van der Weyde et al., 2023). Given the lack of difference in traits between Dryandra populations, we suspect that exposure to one type of mammalian predator has sustained general anti‐predator traits in the Dryandra havened population, which may allow these individuals to respond appropriately to a suite of mammalian predators (i.e. the ‘Multipredator Hypothesis’, Blumstein, 2006). Nonetheless, it is important that this assumption be properly tested.

Our findings contribute to an improved mechanistic understanding of the loss of anti‐predator traits from havened populations. We provide evidence to suggest that consistent exposure to native mammalian predators may be a useful tool for the management of havened populations to ensure they remain viable for release ‘beyond the fence’. With strategic management of Australia's conservation havens, potential perverse outcomes should be avoidable. Specifically, we advocate for the incorporation of behavioural monitoring and management of fauna that are able to persist within havens to maintain critical anti‐predator traits and inform potential triggers for management intervention. This can help ensure that havens continue to benefit conservation as both insurance populations and suitable sources for conservation translocations.

## AUTHOR CONTRIBUTIONS

**Natasha D. Harrison:** Conceptualization (equal); data curation (equal); formal analysis (equal); funding acquisition (equal); writing– original draft (equal); writing – review and editing (equal). **Ben L. Phillips:** Conceptualization (equal); formal analysis (equal); funding acquisition (equal); project administration (equal); resources (equal); supervision (equal); writing – review and editing (equal). **Adrian F. Wayne:** Conceptualization (equal); project administration (equal); resources (equal); supervision (equal); writing – review and editing (equal). **Nicola J. Mitchell:** Conceptualization (equal); project administration (equal); resources (equal); supervision (equal); writing – review and editing (equal).

## CONFLICT OF INTEREST STATEMENT

The authors declare no conflicts of interest.

## Supporting information


Table S1:

## Data Availability

Data and code relevant to this study is available from: https://github.com/natasha‐harrison/Woylie.
