# Supplementary material for: Sustained predation pressure may prevent the loss of anti‐predator traits from havened populations
Source: Ecol Evol. 2024 Jul 10;14(7):e11668. doi: 10.1002/ece3.11668 (PMC11236428; doi:10.1002/ece3.11668)
Supplement: Supplementary file 1 — Table S1: [file ECE3-14-e11668-s001.docx]

**Table S1.** Levels of mammalian predator activity and woylie density among sites during the study.

|  | **Site** | **Haven status** | **Fox** | **Cat** | **Chuditch** | **Woylies** | **Data source** |
| --- | --- | --- | --- | --- | --- | --- | --- |
|  |  |  | mean daily (24h) detections: mdd (95% CI) | | | density estimates per hectare from SECR analyses: ha^-1^ (95% CI) |  |
| Dryandra | Dryandra main block (Village S and Village EW) | Non-havened | 60 cameras (Reconyx PC 900) evenly placed through landscape off-track, 750 m spacing, 30 cm off ground, October 2022 | | | 75 cage traps, along transect at 200 m spacing, open for four nights, May 2022. Estimated by multiplying trap success by a coefficient of 0.0252(0.02066 LCL, 0.02972 UCL) (Wayne et al. 2013a). | DBCA Wheatbelt Region & Biodiversity and Conservation Science, unpublished data |
|  |  |  | 0.032 mdd  (0 - 0.121) | 0.032 mdd  (0 - 0.121) | 0.129 mdd  (0 - 0.297) | 1.390 ha^-1^  (1.14-1.64) |  |
|  | Dryandra Numbat Woylie Sanctuary (DNWS) | Havened | 20 cameras (Reconyx PC 900) evenly placed through landscape off-track, ~750 m spacing, 30 cm off ground, October 2022 | | | 64 cage traps, 8x8 grid at 100 m spacing, open for four nights, October 2022. Single session SECR model with HR detection function and 600 m buffer. | DBCA Wheatbelt Region & Biodiversity and Conservation Science, unpublished data |
|  |  |  | 0 mdd | 0 mdd | 0.266 mdd (0.021 - 0.471) | 1.185 ha^-1^  (0.936 - 1.501) |  |
| Upper Warren | Boyicup | Non-havened | 23 cameras (Reconyx Hyperfire 2 and Swift Enduro) at 200 m spacing along roads, 30 cm off ground, facing road at 45° deployed for 3 months between May and July 2022 | | | 50 cage traps, along transect at 200 m spacing, open for four nights, May 2022. Single session SECR model with HN detection function and 600 m buffer. | _1_Harrison unpublished data  _2_DBCA Donnelly District, unpublished data |
|  |  |  | 0.219 mdd (0.153 - 0.284)_1_ | 0.009 mdd (0-0.040)_1_ | 0.102 mdd (0 – 0.259)_1_ | 0.494 ha^-1^  (0.303 - 0.807)_2_ |  |
|  | Moopinup | Non-havened | 23 cameras (Reconyx Hyperfire 2 and Swift Enduro) at 200 m spacing along roads, 30 cm off ground, facing road at 45° deployed for 3 months between May and July 2022 | | | 50 cage traps, along transect at 200 m spacing, open for four nights, May 2022. Single session SECR model with HN detection function and 600m buffer. | _1_Harrison unpublished data  _2_DBCA Donnelly District, unpublished data |
|  |  |  | 0.217 mdd (0.139 - 0.295)_1_ | 0.018 mdd (0 – 0.077)_1_ | 0.063 mdd (0 – 0.191)_1_ | 0.346 ha^-1^  (0.205 - 0.584)_2_ |  |
|  | Perup Sanctuary | Havened | 20 cameras (Reconyx PC 900) at 500 m spacing along roads, 30 cm off ground, deployed for duration of study. | | | 100 cage traps, 10x10 grid at 100 m spacing, open for four nights, April 2022. Multi-session open population JSSAsecrl model with 800 m buffer. | (Harrison *et al.* *in review*) |
|  |  |  | 0 mdd | 0 mdd | 0 mdd | 0.830 ha^-1^  (0.709 - 0.971) |  |

*Note that owing to the difference in camera deployment between Dryandra (off-track) and the Upper Warren (on tracks), the predator activity rates are not directly comparable. Foxes and cats preferentially use roads, and will experience more detections there (Wayne *et al.* 2013b).

**References**

Harrison, N.D., Thorn, S.M., Maxwell, M.A., Ward, C.G., Wayne, J.C., and Wayne, A.F. (*in review*) Insuring woylies (Bettongia penicillata ogilbyi) against extinction: Establishment of Perup Sanctuary. *Wildlife Research*.

Wayne, A.F., Maxwell, M.A., Ward, C., Vellios, C., Ward, B., Liddelow, G.L., Wilson, I., Wayne, J.C., Williams, M.R. (2013a). The importance of getting the numbers right: quantifying the rapid and substantial decline of an abundant marsupial, *Bettongia penicillata*. *Wildlife Research* **40,** 169-183

Wayne, A.F., Maxwell, M.A., Ward, C.G., Vellios, C.V., Wilson, I.J., and Dawson, K.E. (2013b) Woylie Conservation and Research Project: Progress Report 2010–2013. Department of Parks and Wildlife, Perth.
